# Rectal perforation after barium enema: A case report

**DOI:** 10.1002/ccr3.2563

**Published:** 2019-11-22

**Authors:** Josiana de Oliveira Martins Duarte, Paula Maria Lobato Pestana Pereira, Ana Sofia Gonçalves Sobral, João Pedro Rosa de Oliveira, Henrique José Barrelas Rita, José António de Sousa e Costa

**Affiliations:** ^1^ Internal Medicine Service Hospital Litoral Alentejano Santiago Do Cacém Portugal; ^2^ Intensive Care Unit Hospital do Litoral Alentejano Santiago Do Cacém Portugal

**Keywords:** barium enema, colorectal perforation, futile examinations

## Abstract

Colorectal perforation after barium enema it is a rare complication, but has a high mortality rate. With the emergence of endoscopic examinations, barium enemas have fallen into disuse and doctors are less aware of its complications. This case is of the utmost importance as failure to recognize it could be fatal.

## Graphical abstract

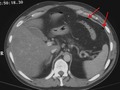

## 1 INTRODUCTION

Barium enema is an old diagnostic examination, safe, and accurate for the study of the colon but, like the majority of invasive techniques, it is necessary to be aware to its complications,^1^ namely allergic reactions to the contrast, perforations of the colon or barium impaction.

Rectal or colonic perforation with intramural dissection or postextravasation of barium for retroperitoneum or peritoneal cavity, although the low incidence (<0.3%)^2^, ^3^ are the most serious complications. However, we question whether this entity is underestimated do to being underdiagnosed.

Although the risk of colon perforation is much lower in barium enema than in colonoscopy (~2%),^3^ mortality rates in the first scenario are higher (as high as 50%)^4^ than those due to colonoscopy, probably due to contrast extravasation and the consequent tissue damage with the enema.

When intraperitoneal contrast extravasation occurs, the situation is especially severe and devastating. The introduction of bacteria and contrast into a cavity, which is otherwise sterile, can cause severe acute peritonitis. In this case with very high mortality rate, aggressive fluid resuscitation^3^ and urgent laparotomy are often required to remove foreign material from the peritoneal cavity, sometimes culminating in colonic ressections.

During the past century, there are a very limited number of cases of colonic perforations following a barium enema, and with this article, we want to highlight the importance of this forgotten entity.

## 2 CASE DESCRIPTION

We report a case of a 72‐year‐old woman who was referred by the Radiology Service to the emergency department with marked neck edema following barium enema. On evaluation, she had no other symptoms, but remained under surveillance for a possible allergic reaction to contrast. There was no improvement after antiallergic treatment. Approximately 24 hours later, the patient begins to present abdominal pain and subcutaneous emphysema of the abdominal, thoracic, and cervical walls.

The patient had a personal history of dyslipidemia, hypothyroidism, gastritis, postischemic stroke status without sequelae, depressive syndrome and anxiety disorder, total hip prosthesis, pulmonary embolism after hip surgery, cholecystectomy, and osteoporosis. No previous history of chronic bowel disease.

The physical examination revealed a distended abdomen with peritoneal reaction in all quadrants. She was febrile (tympanic temperature 38.9°C), hemodynamically stable, with no increase in heart or respiratory rates. She also had subcutaneous emphysema of the abdomen, trunk, and neck.

Routine analytical evaluation (hematology and biochemistry) showed no major changes except for mild leukocytosis with neutrophilia.

**Figure 1:**
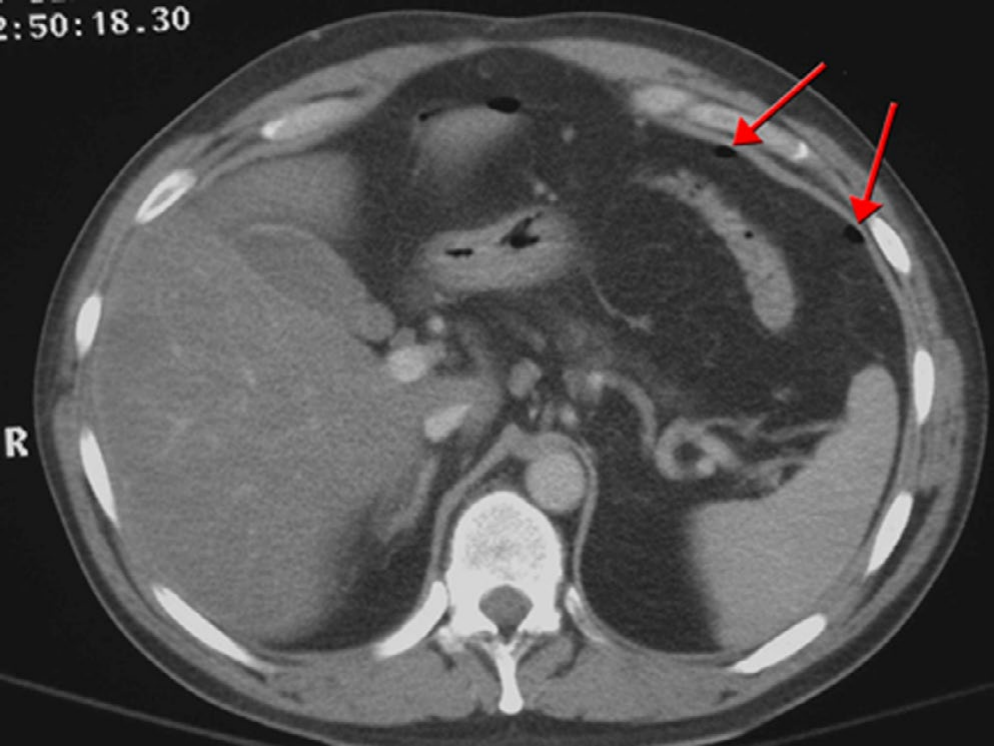
Image from CT scan of abdomen

No signs of pneumoperitoneum were detected on chest radiography. Abdominal computed tomography (Figure 1) revealed gas content filling the abdominal cavity and retroperitoneal region with gas bubbles located at the umbilical region, anterior and posterior para‐renal area and bilateral subphrenic space. Subcutaneous emphysema? Extravasion of contrast?

In this context, the diagnosis of acute abdomen was made and she underwent to surgical intervention. Exploratory laparotomy revealed fluid in the peritoneal cavity and barium leakage into the pelvic cavity. In addition, a perforation of the rectum below the peritoneal reflection was also detected. After an effective abdominal lavage, a colostomy was performed without complications. About 24 hours after surgery, the patient started an oral diet with good tolerance. The remaining postoperative period was uneventful with colostomy functioning normally and resolution of subcutaneous emphysema. The patient was discharged on the 11th postoperative day and referred to surgery appointment (3 weeks after surgery).

It is important to mention that this patient asked her family doctor for this examination, having no symptoms or formal indication for it. As noted earlier in the patient's personal history, she had an anxiety disorder and depressive syndrome with multiple nonspecific complaints, thus asking for another bowel examination to "know if everything was okay."

## 3 DISCUSSION

We report a case of rectal perforation after barium enema, probably due to excessive pressure during the procedure. This case became particularly interesting because of the very anxious nature of the patient, always asking the General Practitioner for multiple examinations (mostly intestinal examinations), although being asymptomatic with recent endoscopic examinations. This time, the doctor complied with the patient's request and prescribed a barium enema without formal indication.

Moreover, in this case, there was a delay in diagnosis, as it was initially interpreted as an allergic reaction rather than a rectal perforation, thus delaying the initiation of treatment.

As with any other invasive examinations and to minimize complications, it is important to perform an assessment of the patient's risk factors prior to the examination, namely history of intestinal inflammation or obstruction, recent endoscopic examination with or without biopsy.^5^ The interval between diagnosis and beginning of treatment in this situation is paramount, as recognizing the symptoms in their early stages will determine the survival of the patient. It is crucial to know how to recognize this situation as early as possible.

Recently, surgical treatment of these perforations has been found to be more persistent and sophisticated, resulting in a more favorable survival of these complications.^6^ However, it is desirable that, in the future, barium enema should be replaced by more sensitive, less invasive and therefore safer techniques, such as computed tomography colonography and magnetic resonance colonography.

There are fewer and fewer reported cases of perforation after barium enemas in recent scientific reports, most likely because colon studies have mostly been replaced by endoscopic examination and that is why we decided to report this case.

It is never too much to remember the old principle, *primum non nocere*, thus avoiding consequences that could have been tragic for this patient.

## CONFLICT OF INTEREST

None declared.

## AUTHOR CONTRIBUTIONS

Josiana Duarte: assumed the responsibility for the publication, making sure that the data are accurate, that all deserving authors have been credited; responsible for data collection and analysis; monitoring the progress of the disease and responsible for the literature review and final approval, submitting revisions and final version, and communicating with editors. Paula Pestana: co‐responsible for data collection and contributed greatly to the case presentation and discussion section.Sofia Sobral: co‐responsible for data collection, contributed greatly to the case presentation. João Oliveira: responsible for the revision of English language. Henrique Rita: responsible for the revision of the article. J. Sousa e Costa: responsible for the revision of the article.
